# Whole-Genome Analysis Reveals Antimicrobial Resistance and Population Structure of Environmental and Veterinary *Acinetobacter baumannii*

**DOI:** 10.3390/ijms27177571

**Published:** 2026-08-24

**Authors:** Saranya Adukkadukkam, Hanka Brangsch, Tamara Kozytska, Sonika Kar, Pavithra Rajkumar, Chandra Sekhar Suppala, P. Anand Kumar, Gamal Wareth, Jayaseelan Murugaiyan

**Affiliations:** 1 Department of Biological Sciences, School of Engineering and Sciences (SEAS), SRM University AP, Amaravathi 522240, Andhra Pradesh, India; saranya_shekharan@srmap.edu.in (S.A.); pavithra_r@srmap.edu.in (P.R.); 2 Friedrich-Loeffler-Institut, Institute of Bacterial Infections and Zoonoses, Naumburger Str. 96a, 07743 Jena, Germany; hanka.brangsch@fli.de (H.B.); tamara.kozytska@fli.de (T.K.); 3 Department of Environmental Biology, Chubu University, Kasugai 4878501, Japan; 4 Department of Biotechnology, REVA University, Bengaluru 560064, Karnataka, India; scsekhar11@gmail.com; 5 Department of Veterinary Microbiology, NTR College of Veterinary Science, Sri Venkateswara Veterinary University, Gannavaram 521102, Andhra Pradesh, India; p_anandkumar@yahoo.com; 6 Institute for Infectious Diseases and Infection Control, Jena University Hospital, Am Klinikum 1, 07747 Jena, Germany

**Keywords:** *Acinetobacter baumannii*, antimicrobial resistance, One Health, pan-genome, core genome MLST, comparative genomics, virulence gene, population genomics

## Abstract

*Acinetobacter* (*A.*) *baumannii* is an important multidrug-resistant pathogen increasingly recognized across animal and environmental settings, and carbapenem-resistant *A. baumannii* (CRAB) is classified as a critical-priority pathogen by the World Health Organization. This study investigated the antimicrobial resistance (AMR) and genomic characteristics of 122 *A. baumannii* isolates comprising 72 veterinary and 50 environmental isolates collected in Andhra Pradesh, India. Antimicrobial susceptibility testing, whole-genome sequencing (WGS), resistance and virulence gene profiling, multilocus sequence typing (MLST), core-genome analysis, single nucleotide polymorphism (SNP) phylogeny, and pan-genome analysis were performed. Overall, 58.2% of isolates were multidrug-resistant (MDR), and 41.8% were extensively drug-resistant (XDR). Sequence type (ST) 52 predominated among veterinary isolates, whereas ST2 was more frequent among environmental isolates. The presence of carbapenem-resistant isolates along with the ST2 lineage enhances the similarity to clinical *A. baumannii*. Several intrinsic resistance genes, including *blaOXA*-23, *armA*, *aph*(*3*″)*-Ib*, *aph*(*6*)*-Id*, *tet*(*B*), *mph*(*E*), and *msr*(*E*), were more prevalent in the ST2-associated population. Virulence-associated determinants were widely conserved. Core-genome MLST (cgMLST) and core-genome SNP (cgSNP) analyses identified highly related isolates within both lineages, while pairwise SNP differences were 0–7. Pan-genome analysis identified 4204 gene clusters and distinct accessory gene patterns between ST2 and ST52. These findings indicate that resistance gene distribution was closely associated with lineage structure and support integrated genomic surveillance of *A. baumannii* across animal and environmental reservoirs.

## 1. Introduction

The rapid emergence and global dissemination of multidrug-resistant (MDR) *Acinetobacter* (*A.*) *baumannii* have become a major public health concern [1]. Recognized by the World Health Organization (WHO) as a critical priority pathogen [2], *A. baumannii* has evolved into one of the most problematic opportunistic bacterial pathogens associated with healthcare settings because of its remarkable ability to survive under adverse environmental conditions and rapidly acquire resistance to multiple antimicrobial agents. The organism is responsible for a wide spectrum of healthcare-associated infections, including ventilator-associated pneumonia, bloodstream infections, urinary tract infections, surgical-site infections, and wound infections, particularly among critically ill and immunocompromised patients [3]. Despite advances in infection prevention and antimicrobial stewardship, infections caused by MDR *A. baumannii* continue to increase worldwide, contributing substantially to prolonged hospitalization, increased healthcare costs and poor clinical outcomes.

The burden of *A. baumannii* infection is particularly alarming in India, where antimicrobial resistance has emerged as a defining characteristic of circulating clinical isolates. Recent surveillance studies indicate that *A. baumannii* accounts for approximately one-fifth of hospital-acquired pneumonia cases, particularly in intensive care units, while carbapenem resistance frequently exceeds 70% and more than 90% of clinical isolates are reported to be carbapenem resistant [4,5]. Consequently, carbapenem-resistant *A. baumannii* (CRAB) has become one of the leading causes of ventilator-associated pneumonia and other severe nosocomial infections across the country [6]. The public health consequences of antimicrobial resistance extend beyond individual healthcare settings, with 61.5% of hospitalized children in six Indian hospitals reported to receive at least one antimicrobial agent, highlighting the need for more appropriate use of antibiotics [7]. Numerous molecular epidemiological studies have further demonstrated clonal dissemination of MDR *A. baumannii* within healthcare facilities, highlighting the urgent need for continuous surveillance and improved understanding of its transmission dynamics [8,9].

Although *A. baumannii* is traditionally regarded as a hospital-associated pathogen, increasing evidence indicates that its ecological distribution extends far beyond healthcare environments. MDR *A. baumannii* isolates have been identified in livestock, poultry, companion animals and a variety of environmental niches, suggesting that these reservoirs may contribute to the persistence and dissemination of antimicrobial resistance. Previous studies have demonstrated that veterinary isolates frequently harbor antimicrobial determinants [10,11,12,13]. Environmental contamination through hospital wastewater, agricultural runoff and domestic sewage has facilitated the recovery of CRAB from rivers, wastewater treatment plants and agricultural soils [14]. These findings support the One Health concept, which recognizes the interconnectedness of human, animal, and environmental health in the evolution and spread of AMR. Among environmental matrices, river ecosystems are of particular importance because they receive inputs from hospitals, livestock farms, municipal sewage and industrial discharge, thereby serving as convergence points for resistant bacteria and antimicrobial resistance genes. Seasonal variations, including monsoon-associated runoff and reduced water flow during dry periods, further influence bacterial dissemination and resistance dynamics [15]. The environment may serve as a reservoir for AMR determinants. Comparing the prevalence and distribution of *A. baumannii* among animal and environmental reservoirs is important for understanding the transmission possibility of the pathogen.

The remarkable adaptability of *A. baumannii* is largely attributable to its genome plasticity. The bacterium possesses a single circular chromosome of approximately 3.5–4.0 Mb together with an extensive accessory genome that is continually reshaped through horizontal gene transfer, homologous recombination and the activity of insertion sequences and other mobile genetic elements [16,17]. This genomic flexibility enables rapid acquisition of AMR determinants, virulence-associated genes and metabolic traits that enhance survival in both clinical and environmental settings. In addition, biofilm formation, persistence under desiccation and the development of persister cells further contribute to its ability to colonize diverse ecological niches. As a result, *A. baumannii* exhibits an open pan-genome, facilitating continuous adaptation to changing selective pressures, particularly those imposed by antimicrobial use [18].

WGS has transformed the understanding of *A. baumannii* epidemiology by enabling high-resolution characterization of strain diversity, resistance mechanisms, and transmission pathways. Comparative genomic analyses have identified several high-risk international clones, of which IC1 and IC2 are the most widely disseminated and are responsible for numerous hospital outbreaks worldwide [19,20]. IC2 predominates across much of Asia and has been strongly associated with carbapenem resistance [21]. Studies from India indicate that IC1 remains the predominant lineage, although IC2, IC7 and IC8 have also been reported. ST2 is an internationally disseminated lineage associated with multidrug resistance and hospital outbreaks, whereas ST52 has been reported from both clinical and non-clinical sources [22,23,24]. The geographical variation in clone distribution highlights the importance of regional genomic surveillance for understanding the evolution and dissemination of high-risk lineages and for informing infection control and antimicrobial stewardship strategies.

Despite the increasing availability of genomic data from clinical isolates, comparatively little is known about the diversity, AMR profiles, and clonal composition of *A. baumannii* circulating in environmental and veterinary reservoirs in India. Such information is essential for understanding the environmental persistence of high-risk clones and their potential contribution to the transmission of clinically important AMR. Therefore, the present study aimed to characterize the genomic diversity, AMR determinants, and population structure of *A. baumannii* isolates recovered from environmental and veterinary sources in Guntur and Prakasam districts of Andhra Pradesh, India, using WGS and comparative genomic analyses. We hypothesize that *A. baumannii* circulating among environmental and veterinary sources harbors clinically relevant AMR determinants and virulence-associated genes. This study provides baseline genomic surveillance data within a One Health framework and contributes to a better understanding of the circulation of multidrug-resistant *A. baumannii* beyond hospital settings.

## 2. Results

### 2.1. Antimicrobial Susceptibility Profiles and Phenotypic Resistance Mechanisms of A. baumannii Isolates

**Figure 1:**
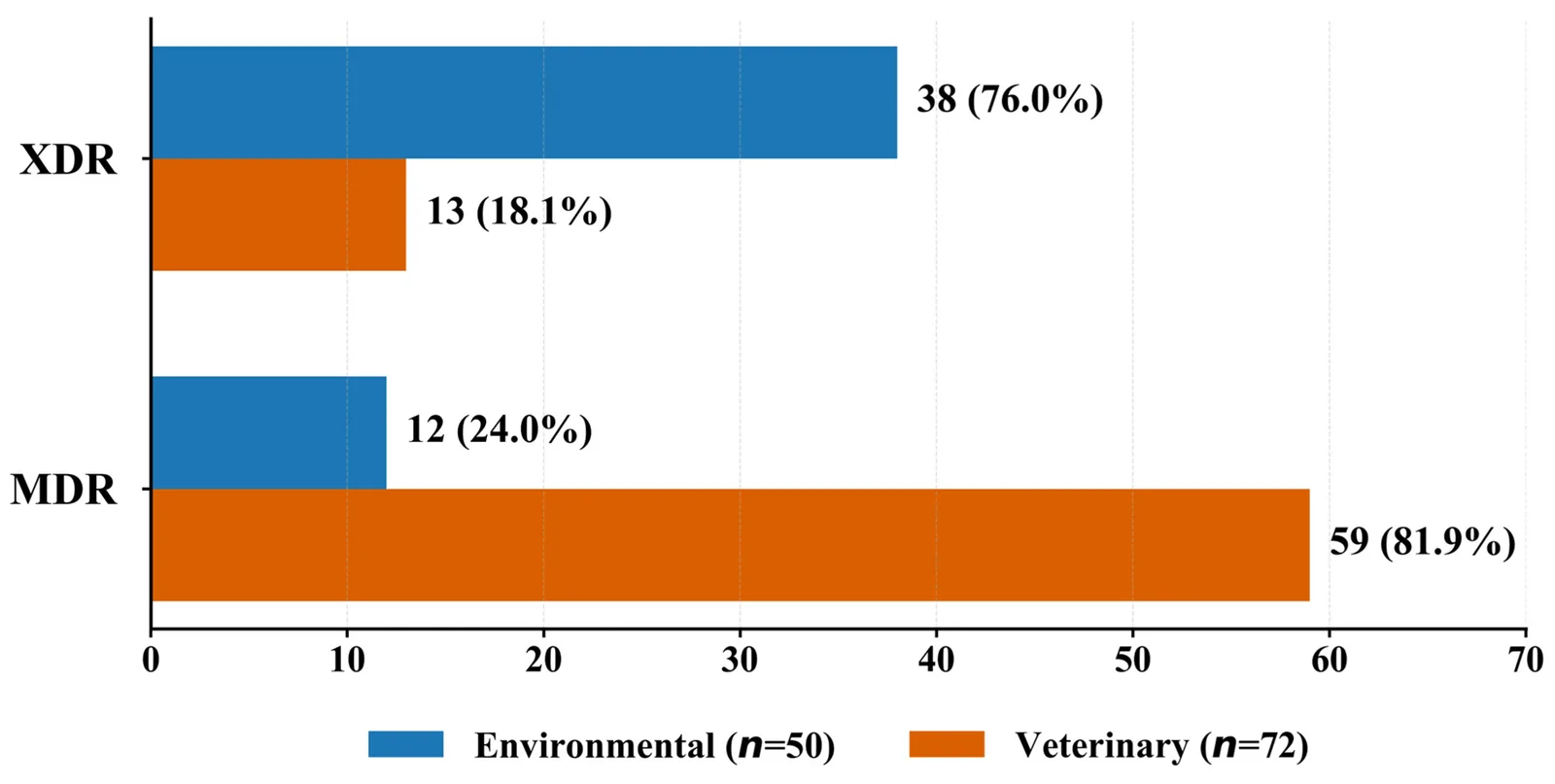
Distribution of MDR and XDR phenotypes among environmental and veterinary *A. baumannii* isolates. MDR, multidrug-resistant; XDR, extensively drug-resistant.

A total of 122 non-duplicated *A. baumannii* isolates, comprising 72 veterinary and 50 environmental isolates, were subjected to AST. Overall, 71 (58.2%) isolates were classified as multidrug-resistant (MDR), and 51 (41.8%) isolates as extensively drug-resistant (XDR). No pan-drug-resistant (PDR) isolates were identified. MDR was the predominant phenotype among veterinary isolates, accounting for 59/72 (81.9%) isolates, whereas XDR was more frequently observed among environmental isolates, with 38/50 (76.0%) isolates classified as XDR (Figure 1; Table S2).

The distribution of MDR and XDR phenotypes differed significantly between veterinary and environmental isolates (two-sided Fisher’s exact test, OR = 14.37, 95% CI: 5.94–34.79, *p* < 0.0001), with veterinary isolates showing significantly higher odds of an MDR rather than XDR phenotype compared with environmental isolates.

The AST profiles of the 122 isolates are summarized in Table 1. Resistance was observed across multiple antimicrobial classes in both veterinary and environmental isolates. Complete resistance was detected against chloramphenicol (CMP), fosfomycin (FOS), piperacillin (PIP), and cefotaxime (CTX), with all isolates categorized as resistant. High resistance was also observed for trimethoprim/sulfamethoxazole (80.3%), followed by ciprofloxacin (41.0%), levofloxacin (42.6%), amikacin (40.2%), ceftazidime (42.6%), ceftazidime/avibactam (41.8%), ceftolozane/tazobactam (41.0%), imipenem (43.4%), and meropenem (41.0%). Among the antimicrobial agents tested, tigecycline showed MICs ≤ 0.25 µg/mL in 71 isolates, while the remaining isolates had MICs ranging between 0.5 and 2 µg/mL. Colistin demonstrated predominantly intermediate susceptibility with MIC values of ≤2 µg/mL.

Source-stratified analysis showed marked differences in AMR profiles. Environmental isolates showed significantly higher resistance to meropenem, levofloxacin, imipenem, amikacin, ciprofloxacin, tigecycline, ceftazidime/avibactam, ceftazidime, piperacillin/tazobactam, and ceftolozane/tazobactam than veterinary isolates (FDR-adjusted *p* < 0.0001 for all comparisons). In contrast, trimethoprim/sulfamethoxazole resistance was significantly higher among veterinary isolates (98.6% vs. 54.0%; OR = 60.48, 95% CI: 7.78–470.08; FDR-adjusted *p* < 0.0001). No significant source-associated differences were observed for colistin, chloramphenicol, fosfomycin, cefotaxime, or piperacillin. Complete antimicrobial-specific statistical comparisons are provided in Supplementary Material S2.

Resistance categories were assigned according to the international consensus definitions proposed by Magiorakos et al., [25]. In brief, isolates were classified as multidrug-resistant (MDR) when they exhibited non-susceptibility to at least one agent in three or more antimicrobial categories. The antimicrobial categories included in the analysis were (i) penicillins plus β-lactamase inhibitors (piperacillin/tazobactam), (ii) extended-spectrum cephalosporins (ceftazidime), (iii) carbapenems (imipenem and meropenem), (iv) fluoroquinolones (ciprofloxacin and levofloxacin), (v) aminoglycosides (amikacin), (vi) polymyxins (colistin), (vii) glycylcyclines (tigecycline), and (viii) folate pathway inhibitors (trimethoprim/sulfamethoxazole). Phenotypic detection agents (e.g., ceftazidime/3-APB, meropenem/EDTA, meropenem/3-APB, temocillin) and antimicrobial agents without established categories in the Magiorakos definitions were not considered for MDR classification. The isolates were considered extensively drug-resistant (XDR) when they showed non-susceptibility to at least one agent in all but two or fewer antimicrobial categories (i.e., bacterial isolates remained susceptible to only one or two categories).

### 2.2. Genome Sequencing and AMR Determinants

Genome assembly of the 122 *A. baumannii* isolates had a mean genome size of 3.94 Mb and exhibited a mean GC content of 39%. The average sequencing depth was 358× (range: 76×–731×) and contained an average of 3704 coding sequences (CDSs) per isolate. Genome assembly characteristics were compared between veterinary and environmental isolates. No statistically significant differences were observed in genome assembly size (median: 3,957,653 bp vs. 3,952,827 bp; Mann–Whitney U test, *p* = 0.776), number of contigs (*p* = 0.726), N50 values (*p* = 0.711), or GC content (*p* = 0.202), indicating comparable genome assembly characteristics between the two isolate sources.

**Figure 2:**
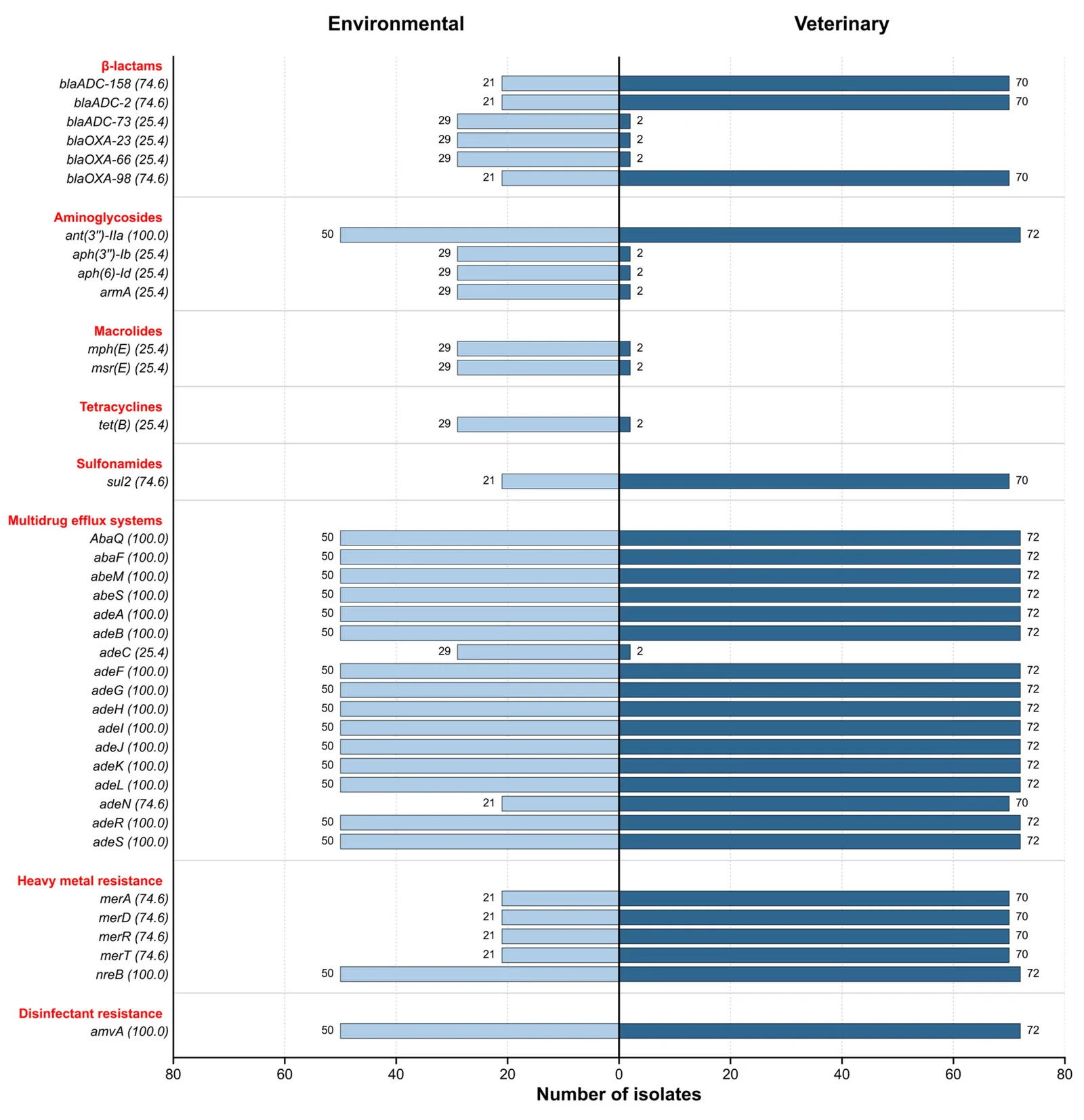
Distribution of AMR determinants among veterinary and environmental *A. baumannii isolates*. The prevalence of AMR genes associated with β-lactam, aminoglycoside, macrolide, tetracycline, and sulfonamide resistance, together with intrinsic multidrug efflux systems, heavy metal resistance genes, and disinfectant-associated resistance determinants identified by whole-genome sequencing. Values on the bars indicate the number of isolates carrying each gene.

Genome analysis identified AMR genes associated with β-lactams, aminoglycosides, tetracyclines, sulfonamides, and macrolides, together with multiple intrinsic resistance determinants. Genes encoding multidrug efflux pumps, disinfectant tolerance, and heavy metal resistance were also widely distributed across the collection. The complete distribution of all detected resistance genes and their associated resistance mechanisms is presented in Table S3, while the prevalence of clinically relevant resistance genes among veterinary and environmental isolates is shown in Figure 2.

Among the intrinsic β-lactam resistance determinants, *blaADC-158* and *blaOXA-98* were detected in 91 (74.6%) isolates, comprising 70 veterinary and 21 environmental isolates. In contrast, *blaADC-73*, *blaOXA-23*, and *blaOXA-66* were identified in 31 (25.4%) isolates, including 29 environmental and two veterinary isolates. Similarly, the aminoglycoside resistance genes *armA*, *aph*(*3*″)*-Ib*, *aph*(*6*)*-Id*, together with the tetracycline resistance gene *tet*(*B*) and the macrolide resistance genes *mph*(*E*) and *msr*(*E*), were also predominantly detected among environmental isolates. The sulfonamide resistance gene *sul2* was identified in 91 (74.6%) isolates and showed a distribution similar to that of *blaADC-158* and *blaOXA-98.* In addition to acquired AMR genes, all isolates carried multiple intrinsic resistance determinants, including genes encoding resistance-nodulation-division (RND) efflux systems (*adeABC*, *adeFGH*, *adeIJK*), the MATE- family transporters *abeM* and *AbaQ*, the multidrug efflux regulator *abeS*, the fosfomycin resistance gene *abaF*, the disinfectant-associated efflux gene *amvA*, and heavy metal resistance genes of the *mer* operon (*merA*, *merD*, *merR*, and *merT*) together with *nreB*. The complete distribution of these genes is provided in Table S3. The further classification of ARGs based on their chromosomal or plasmid origin is detailed in Table S4.

To statistically evaluate source-associated differences in antimicrobial resistance determinants, the prevalence of individual resistance genes was compared between veterinary and environmental isolates using two-sided Fisher’s exact tests with Benjamini–Hochberg false discovery rate (FDR) correction. Nineteen resistance genes differed significantly between the two isolate sources after FDR adjustment (adjusted *p* < 0.05), whereas the remaining genes showed no significant differences. Genes including *blaADC-158*, *blaOXA-98*, *blaADC-2*, *sul2*, *adeN*, and the mercury resistance genes (*merA*, *merD*, *merR*, and *merT*) were significantly more prevalent among veterinary isolates, whereas *blaOXA-23*, *blaOXA-66*, *armA*, *aph*(*3*″)*-Ib*, *aph*(*6*)*-Id*, *tet*(*B*), *mph*(*E*), and *msr*(*E*) were significantly more frequent among environmental isolates. Complete statistical results, including odds ratios, 95% confidence intervals, raw *p*-values, and FDR-adjusted *p*-values, are provided in Supplementary Material S2.

Chromosomal point mutations associated with AMR were detected using AMRFinderPlus. Three mutations were identified: *ftsI_A515V*, which encodes a substitution in penicillin-binding protein 3 (PBP3); *parC_S84L*, which is associated with fluoroquinolone resistance; and *gyrA_S81L*, which results in a serine-to-leucine substitution at position 81 of the DNA gyrase subunit A protein and is associated with fluoroquinolone and quinolone resistance.. These mutations were identified in 31 (25.4%) isolates, whereas the remaining 91 (74.6%) isolates lacked these substitutions. All three mutations co-occurred in the same isolates (Table S5).

### 2.3. Virulence-Associated Genes

Whole-genome sequencing identified a total of 120 virulence-associated genes representing multiple functional categories involved in the pathogenicity and environmental persistence of *A. baumannii*. These included genes associated with biofilm formation, iron acquisition, pili assembly, lipopolysaccharide biosynthesis, type II and type VI secretion systems, phospholipase production, quorum sensing, and other virulence-related functions.

Many virulence-associated genes were highly conserved across the isolate collection. Genes involved in lipopolysaccharide biosynthesis (*lpxA*, *lpxB*, *lpxC*, *lpxD*, *lpxL*, *lpxM*, and *lpsB*), biofilm formation (*bap* and the *csu* operon), iron acquisition (*bas-ABCDEFGHIJ* and *bau-ABCDEF*), type II secretion (*gspC-O*), type VI secretion (*tss-ABCDEFGHIJKLM*, *hcp*, *vgrG*, and *clpV*), and phospholipase production (*plc1*, *plc2*, and *plcD*) were detected in nearly all isolates irrespective of their source.

**Figure 3:**
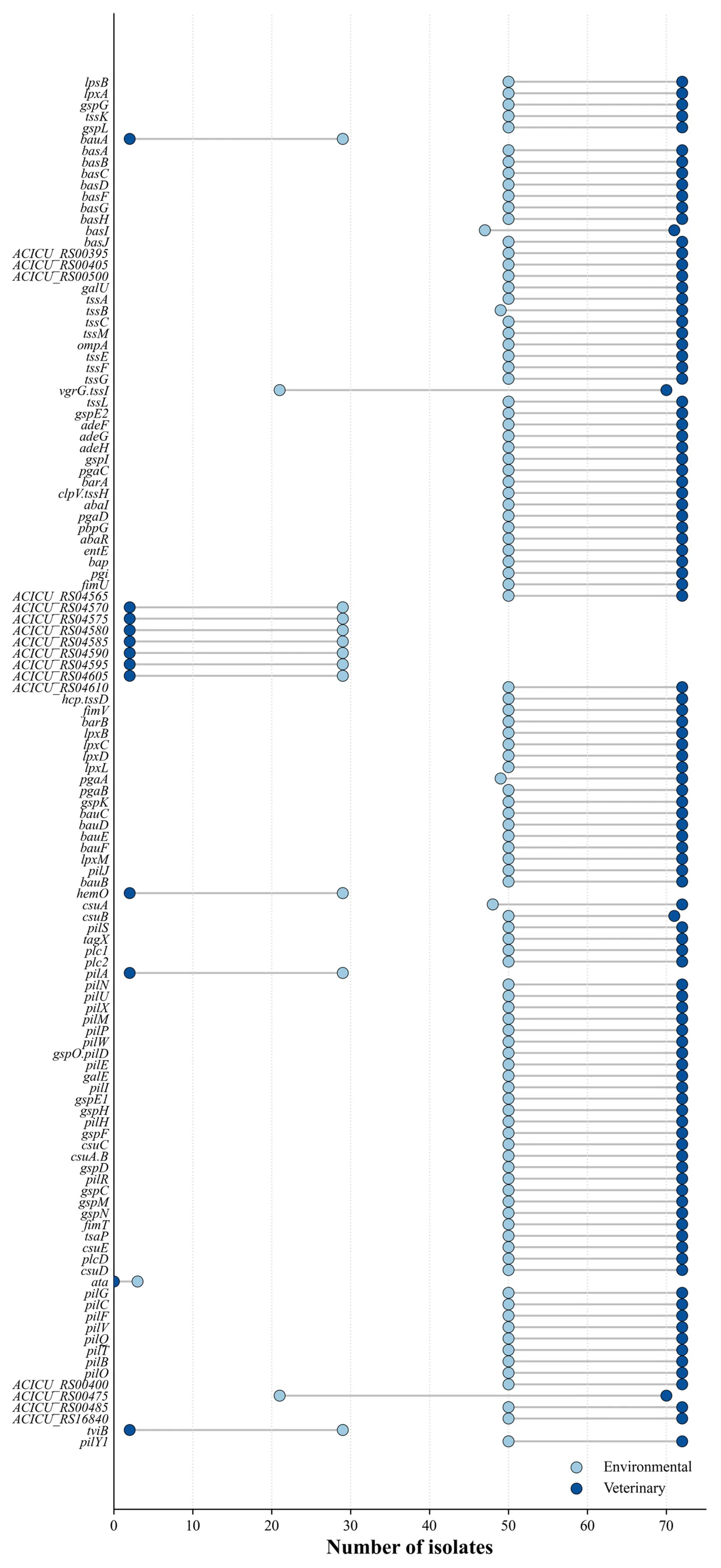
Distribution of virulence-associated genes among veterinary and environmental *A. baumannii* isolates. The prevalence of virulence-associated genes is shown for veterinary (dark blue) and environmental (light blue) isolates. Horizontal connecting lines illustrate differences in gene prevalence between the two isolate groups. Most virulence-associated genes were highly conserved across both populations.

Comparative analysis between veterinary and environmental isolates revealed only limited variation in virulence gene distribution. Most differences were confined to a small subset of genes, whereas the core virulence genes remained conserved between the two groups of isolates (Figure 3), and the location of each VFG is given in Table S6.

Of the 120 virulence genes examined, 109 (90.8%) showed no significant difference in prevalence after Benjamini–Hochberg FDR correction. Only 11 genes, including *bauA*, *pilA*, *tviB*, *hemO*, and seven locus-tagged genes, showed significant source-associated differences (FDR-adjusted *p* < 0.0001). These genes were detected in 58.0% of environmental isolates but only 2.8% of veterinary isolates. Despite this broad conservation at the individual-gene level, overall virulence gene composition differed significantly between the two source populations (PERMANOVA, pseudo-F = 75.45, *p* = 0.0001; 9999 permutations). Complete statistical results are provided in Supplementary Material S2.

### 2.4. Sequence Typing and Population Structure

MLST analysis based on the Pasteur scheme identified only two STs among the 122 isolates, ST2 and ST52. ST52 was the predominant lineage, comprising 91/122 (74.6%) isolates, whereas ST2 accounted for the remaining 31/122 (25.4%) isolates. A marked difference in lineage distribution was observed between veterinary and environmental sources. Among veterinary isolates, ST52 represented 70/72 (97.2%) isolates, while only two isolates (2.8%) belonged to ST2. In contrast, environmental isolates consisted predominantly of ST2 (29/50, 58.0%), whereas ST52 accounted for 21/50 (42.0%) isolates. These findings demonstrate a strong association of ST52 with veterinary isolates and ST2 with environmental isolates.

**Figure 4:**
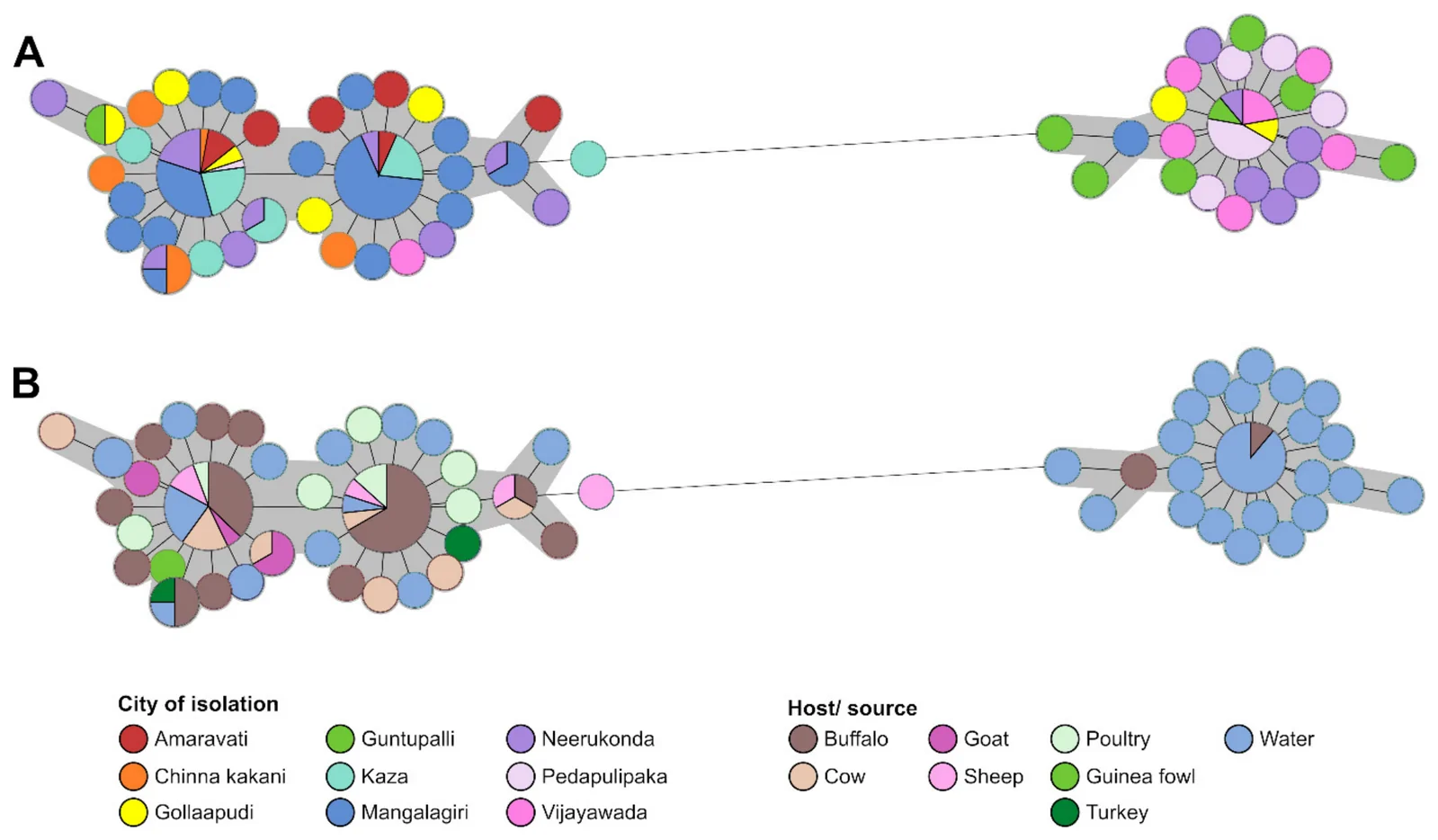
Minimum spanning tree of the 122 *A. baumannii* isolates based on cgMLST profiles. (**A**) shows a panel of isolates sharing identical cgMLST profiles, colored according to the city of isolation (geographical location). (**B**) shows a panel of the same isolates with identical cgMLST profiles, colored according to the host/source of isolation. Each circle represents one or more isolates sharing an identical cgMLST profile, with circle size proportional to the number of isolates. Connecting lines indicate allelic relationships between isolates, and the pie charts within each node represent the distribution of isolates according to the corresponding metadata category. Five major cgMLST clusters were identified using a threshold of nine allelic differences, whereas four isolates remained as singletons outside the defined clusters.

To further investigate the genetic relatedness of the isolates, cgMLST was performed using the Ridom *A. baumannii* cgMLST scheme. Applying a cluster threshold of nine allelic differences grouped the 122 isolates into five major clusters, while four isolates remained as singletons (Figure 4). The maximum allelic distance observed across the collection was 2237 alleles, indicating substantial genomic diversity.

The minimum spanning tree revealed that isolates clustered primarily according to sequence type, with ST52- and ST2-associated isolates forming distinct genetic groups. Most veterinary isolates were distributed within the dominant ST52 clusters, whereas environmental isolates were largely associated with the ST2 lineage. Within each lineage, several isolates shared identical cgMLST profiles, while others differed by only a few core-genome alleles, indicating the presence of closely related isolates across multiple animal hosts and environmental sampling sites (Figure 4).

### 2.5. Core-Genome SNP Phylogeny and SNP Differences

Core-genome SNP analysis was performed separately for ST2 and ST52 isolates to assess within-lineage genomic relatedness. Pairwise SNP differences ranged from 0 to 7 SNPs in both ST-lineages. Within ST2, many isolates showed no detectable pairwise SNP differences, while closely related groups were separated by a small number of SNPs. A similar pattern was observed among ST52 isolates, also indicating limited within-lineage variation.

**Figure 5:**
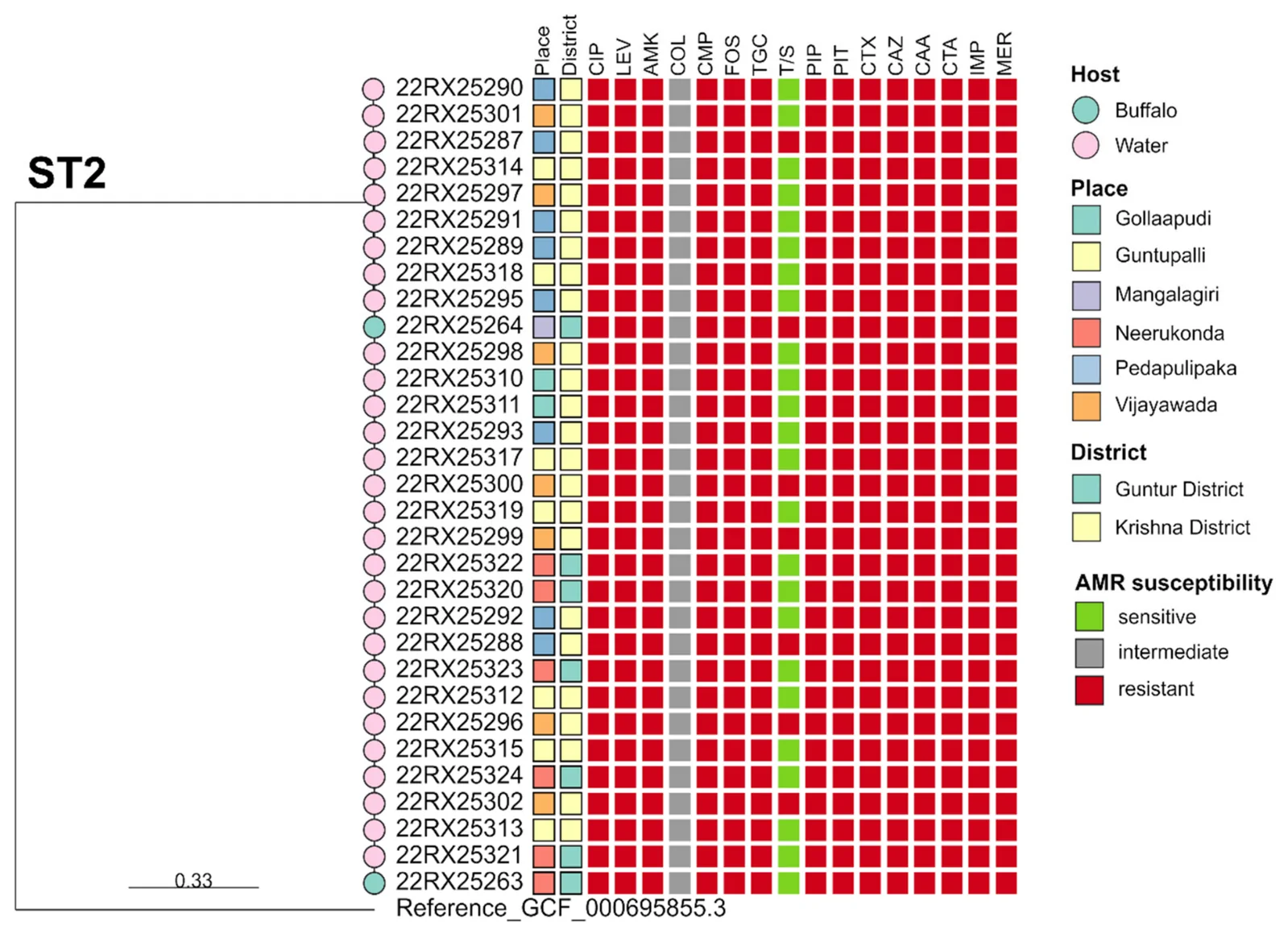

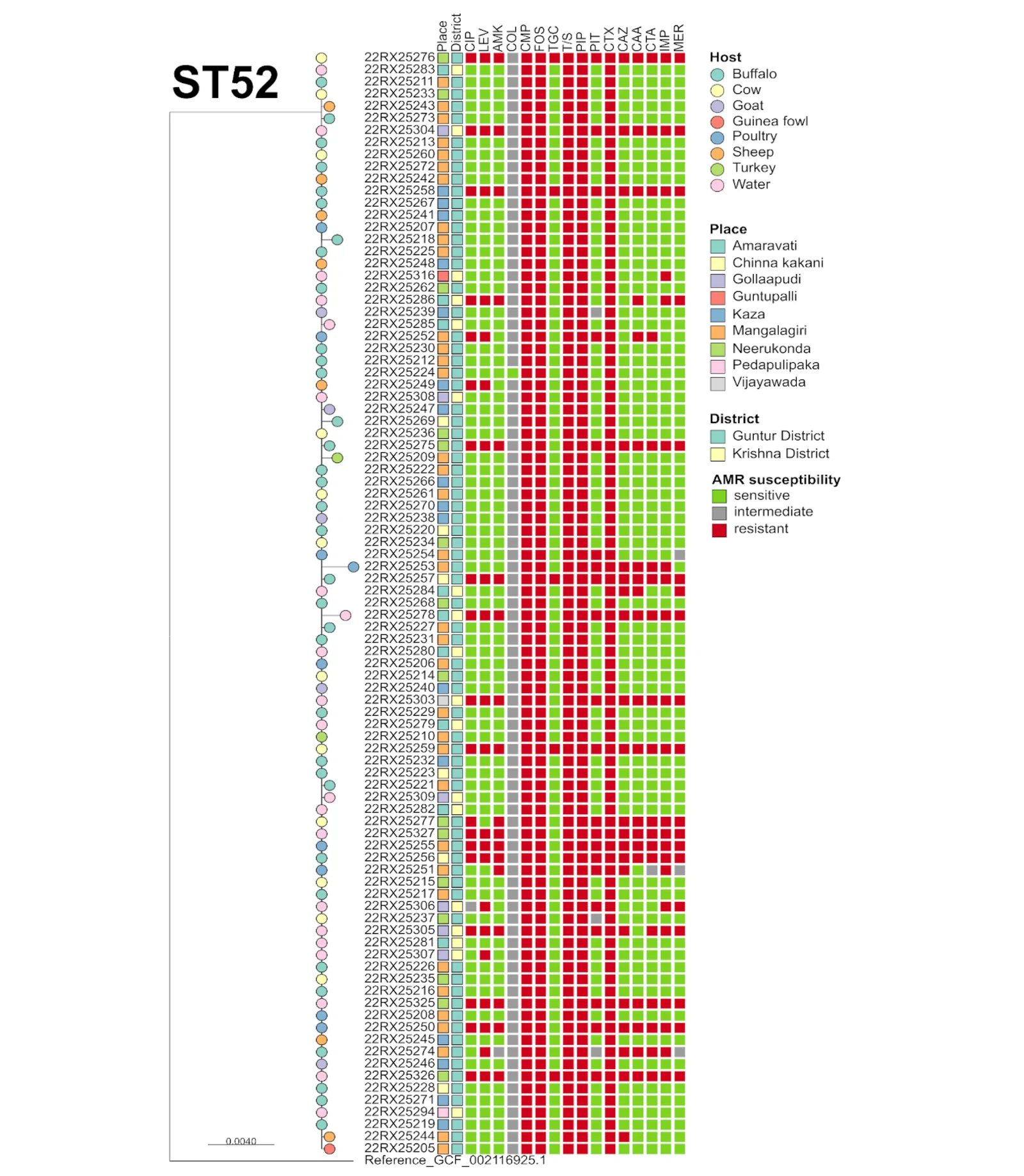
Core-genome SNP-based phylogenetic relationships among ST52 and ST2 *A. baumannii* isolates.

The Maximum-likelihood phylogenetic analysis further showed close clustering of isolates within each ST (Figure 5). ST52 comprised isolates from diverse veterinary hosts as well as with environmental isolates. Isolates from different sources occurred within the same phylogenetic groups, and several showed identical or near-identical SNP profiles. The ST2 phylogeny was dominated by environmental isolates, consistent with the source distribution identified by MLST analysis, with the two veterinary ST2 isolates also positioned within this lineage.

Overall, the SNP analysis identified groups of highly similar isolates within both ST2 and ST52, including isolates recovered from different hosts and environmental sources.

Maximum-likelihood phylogenetic trees were constructed separately for ST52 and ST2 isolates based on core-genome SNP alignments. Isolates are indicated by colored circles according to the host or environmental source of isolation. The adjacent annotation tracks indicate isolate metadata as shown in the figure legend. Branch lengths represent the number of nucleotide substitutions per site.

### 2.6. Pan-Genome Analysis

A pan-genome analysis of 122 *A. baumannii* genomes identified a total of 4204 gene clusters. Of these, 3109 (73.9%) were classified as core genes and were present in ≥99% of the isolates. The soft core-genome comprised 45 genes (1.07%) present in 95–<99% of isolates, while 1049 genes (24.9%) constituted the shell genome and were detected in 15–< 95% of the isolates. Only one gene was assigned to the cloud genome (<15% of isolates).

**Figure 6:**
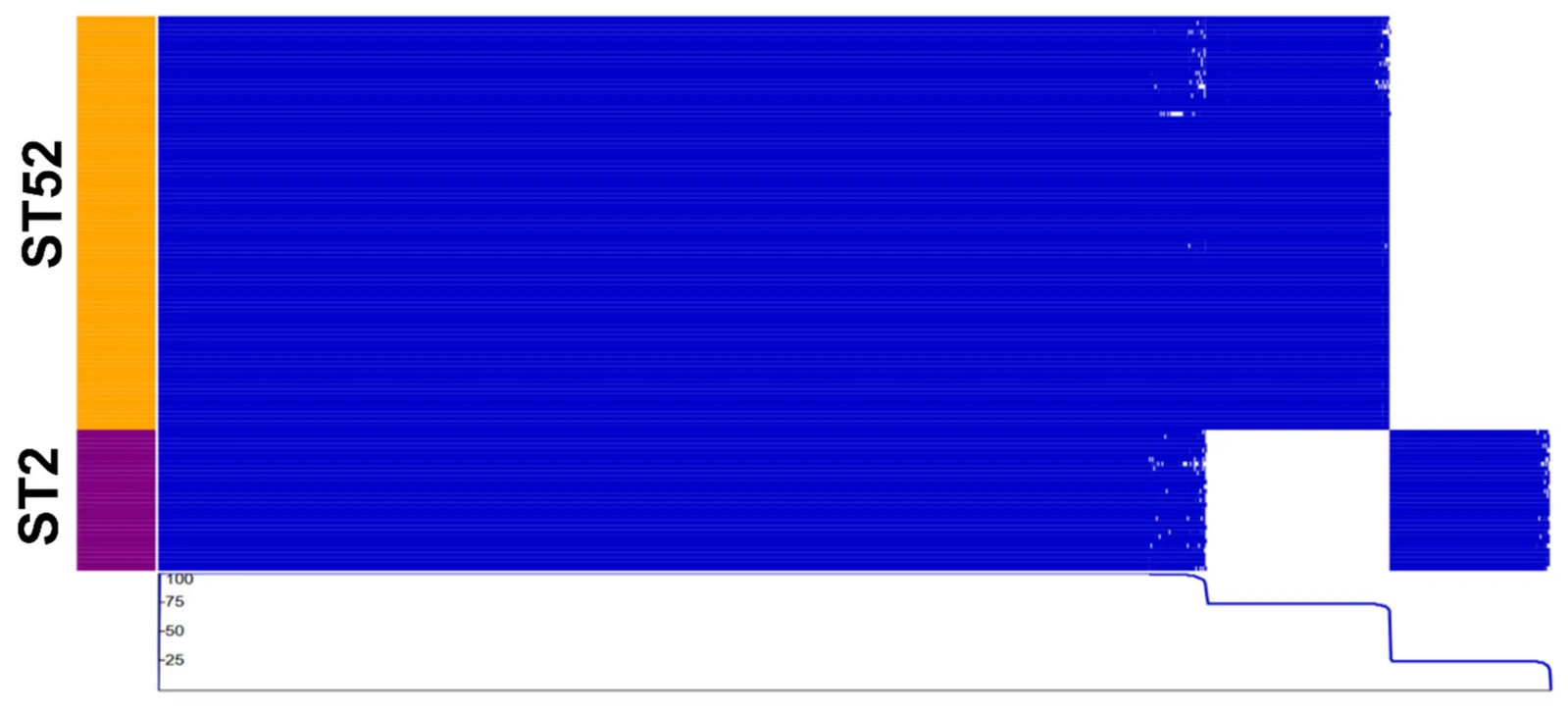
Pan-genome structure and gene presence–absence patterns of the 122 isolates.

The gene presence–absence matrix showed a highly conserved core-genome across the isolate collection, together with variation in accessory gene content (Figure 6). The distribution of accessory genes differed between the two major sequence types, with ST52 and ST2 showing distinct gene presence–absence patterns. This separation was consistent with the population structure identified by MLST, cgMLST, and core-genome SNP analyses.

The phylogenetic tree was constructed from the filtered core gene alignment generated by Panaroo. The gene presence–absence matrix shows the distribution of gene clusters across individual isolates, with blue indicating gene presence and white indicating gene absence. The blue squares on the right indicate genes present only in a subset of isolates. Sequence types are indicated by the annotation bar on the left (orange, ST52; purple, ST2).

### 2.7. Plasmid Prediction and Plasmid-Associated Sequences

Plasmid prediction identified contigs with similarity to previously reported *A. baumannii* plasmids. BLAST (2.16.0+) analysis of the predicted plasmid-associated sequences showed nucleotide identities ranging from 98.7% to 100%, with query coverage ranging from 45% to 100%. The identified matches included plasmids reported in *A. baumannii*, including pMAC of strain ATCC 19606, an unnamed plasmid of strain AR_0052, pAB3927, pATCC19606-1, pB20AB01a, pA85-1b, and pG20AB05, as well as pSGAir0122 from *A. schindleri*.

Several plasmid-associated sequence matches were detected in ST52 isolates recovered from different veterinary hosts and environmental water samples. Plasmid matches associated with ST2 isolates were recovered from buffalo and environmental water samples. The distribution and closest BLAST matches of the predicted plasmid-associated contigs are provided in Table S7.

## 3. Discussion

*A. baumannii* remains one of the most important AMR pathogens of One Health importance [26]. Although it is primarily recognized for its significance/pathogenicity in health-associated infections, its occurrence in animals and environmental reservoirs has received increasing attention. Carbapenem-resistant *A. baumannii* is particularly concerning because of the limited therapeutic options and poor clinical outcomes associated with these infections [27]. In the present study, *A. baumannii* isolates from veterinary and environmental sources were investigated to compare their antimicrobial resistance profiles and genomic characteristics. Each *A. baumannii* isolate included in the study was treated as an independent observation, as it was recovered from an individual sample and processed independently throughout the isolation and genomic analysis workflow. However, we acknowledge that isolates collected from the same farm, animal, or location may not be epidemiologically independent and could represent the same circulation clone, which is reflected by their identical cgMLST profile and 0–7 SNP variation. The difference in AMR phenotypes between the two sources was also reflected in their lineage distribution.. More than half of the isolates were classified as multidrug-resistant (MDR; 58.2%), while 41.8% exhibited an extensively drug-resistant (XDR) phenotype, and no pan-drug-resistant isolates were identified. MDR was predominant among veterinary isolates, whereas XDR was more frequent among environmental isolates. The occurrence of XDR profiles among environmental isolates may be linked to climate-driven environmental changes, as rising temperatures enhance microbial growth, increase bacterial population densities, and facilitate horizontal gene transfer, thereby promoting the emergence and dissemination of antimicrobial resistance genes [28]. The marked difference in MDR/XDR distribution was statistically supported, with veterinary isolates showing approximately 14-fold higher odds of exhibiting an MDR rather than an XDR phenotype relative to environmental isolates. This finding reinforces the distinct resistance profiles observed between the two source populations.

The phenotypic resistance profiles also showed a good source-associated pattern, with environmental isolates exhibiting significantly higher resistance to multiple antimicrobial classes, including carbapenems, fluoroquinolones, aminoglycosides, and tigecycline. Conversely, trimethoprim/sulfamethoxazole resistance was significantly more frequent among veterinary isolates. These contrasting susceptibility profiles further support distinct resistance patterns between the two source populations. However, given the strong association between source and sequence type, these differences should also be interpreted in the context of the unequal distribution of ST52 and ST2.

The occurrence of resistant *A. baumannii* in aquatic environments is increasingly recognized as a public health concern, particularly in water systems exposed to anthropogenic and agricultural activities [29,30]. Complete resistance to fosfomycin, chloramphenicol, piperacillin, cefotaxime, and ceftazidime/3-APB was observed in the present collection, while resistance to trimethoprim/sulfamethoxazole was also high. Several studies of animal-derived *A. baumannii* have reported retained susceptibility to selected last-line or broad-spectrum antimicrobial agents [31,32]. In contrast, 121 of 122 isolates (99%) are intermediate-resistant to colistin, the last-resort antimicrobial against MDR bacteria including *A. baumannii*. This finding is strikingly different from many clinical studies on CRAB (for which colistin remains the effective drug) [32]. Genomic analysis identified 37 resistance-associated genes representing several resistance mechanisms, including antimicrobial efflux, enzymatic drug inactivation, target modification, and resistance to heavy metals and disinfectants. A prominent feature of the resistome was the widespread occurrence of multidrug efflux-associated determinants. Genes belonging to the *AdeABC*, *AdeFGH*, and *AdeIJK* resistance-nodulation-division efflux systems, together with *abeM*, *AbaQ*, *abeS*, and *abaF*, were highly conserved across the isolate collection. RND efflux systems are important contributors to the intrinsic resistance of *A. baumannii* and can influence susceptibility to several structurally unrelated antimicrobial compounds [33]. The carbapenem resistance in our collection was predominantly associated with intrinsic class D-beta lactamases detected by WGS, including *blaOXA-66* (25.4%), *blaOXA-98* (74.6%), *blaOXA-23* (25.4%), and *blaOXA-497* (74.6%), together with *blaADC* cephalosporinase variants (*blaADC-2* and *blaADC-72*). Thus, the carbapenem resistance in our isolate collection is primarily mediated by *OXA-type* oxacillinase rather than *KPC* or *MBL*-mediated resistance. The widespread occurrence of these determinants among both veterinary and environmental isolates indicates a conserved intrinsic resistance background across the study population.

The genome architecture of the isolates was relatively conserved, with similar GC content across the isolates, indicating the genome stability of *A. baumannii*. There was no major source-specific genome size difference except for the variation in lineage-associated differences. However, the resistance determinants showed a markedly structured distribution. The β-lactamases genes *blaADC-158* and *blaOXA-98*, together with *sul2*, were detected in 74.6% of the isolates and were predominantly represented among veterinary isolates. In contrast, *blaADC-73*, *blaOXA-23*, and *blaOXA-66*, as well as *armA*, *aph*(*3*″)*-Ib*, *aph*(*6*)*-Id*, *tet*(*B*), *mph*(*E*), and *msr*(*E*), were concentrated among environmental isolates. The occurrence of *blaOXA-23* is particularly important because *OXA-23* is a major carbapenemase associated with carbapenem resistance in *A. baumannii* [34]. In our collection, the high prevalence of carbapenem resistance likely reflects both clonal expansion of high-risk lineages (ST2) and diverse resistance determinants. The presence of *blaOXA-23* and intrinsic *blaOXA-51* genes is an indication of dissemination of carbapenem resistance [35]. Excessive use of antibiotics in both environmental and veterinary sectors also increases the persistence of antibiotics among the sources and adaptation of resistance over time. Our study findings on ARGs are consistent with published Indian studies. The high prevalence (98%) of *blaOXA-23*-like in 122 isolates was also reported in 98% of 103 CRAB isolates and 91.7% of 133 isolates as per other Indian Studies [36,37]. The same trend follows for metallo-beta-lactamases, where the *blaNDM-1* gene is reported to be less prevalent, which is in line with the same reference studies. The carbapenem resistance varies over regions in an epidemiological context. The report from Asia-Pacific surveillance conducted from 2012 to 2019, ~71.7% of *A. baumannii* isolates were carbapenem resistant [38]. A Latin American study conducted in the 2015–2025 period identified ~72.6% of CRAB isolates of clinical origin [39]. This emphasizes the epidemiological variation in resistance. The simultaneous occurrence of *armA* and aminoglycoside-modifying enzyme genes further demonstrates the presence of multiple acquired resistance mechanisms in these isolates [40]. The combination of intrinsic efflux-associated determinants and acquired resistance genes may therefore contribute to the broad resistance profiles observed in the isolate collection.

Importantly, the distribution of several intrinsic resistance genes closely paralleled the sequence type distribution observed in this study. The group of 29 environmental and 2 veterinary isolates carrying *blaOXA-23*, *armA*, *aph*(*3*″)*-Ib*, *aph*(*6*)*-Id*, *tet*(*B*), *mph*(*E*), and *msr*(*E*) corresponded to the ST2 population. Conversely, *blaADC-158*, *blaOXA-98*, and *sul2* showed a distribution corresponding to the predominance of ST52. This close correspondence suggests that differences in resistance gene profiles between veterinary and environmental isolates may be strongly influenced by the lineage composition of the two populations rather than by ecological source alone. The association of resistance determinants and resistance islands with dominant *A. baumannii* lineages has also been observed in genomic studies of high-risk clones [41,42]. Thus, source-associated differences in the present collection should be considered together with the underlying population structure.

The concordance between phenotypic AMR pattern and detected AMR genes was observed among the isolates for some antibiotics such as (a) phenotypic β-lactam resistance was observed with the presence of intrinsic β-lactamase genes such as *blaOXA-51* like variants and *blaADC* alleles, (b) detection of aminoglycoside modifying genes (*armA*, *ant*(*3″*)*-IIa*) for the isolates showing resistance to aminoglycosides, (c) sulfonamide and trimethoprime phenotypic resistance was observed with the detection of sulfonamide resistant genes (*sul2*). Point mutations in chromosomal housekeeping genes constitute one of the principal mechanisms underlying AMR in *A. baumannii*, particularly resistance to fluoroquinolones and β-lactam antibiotics [43]. The *gyrA_S81L* substitution is one of the best-characterized quinolone resistance-determining region (QRDR) mutations in *A. baumannii* [44]. Similarly, the *parC_S84L* mutation alters topoisomerase IV, further decreasing fluoroquinolone susceptibility [45]. The *ftsI_A515V* mutation occurs in the gene encoding penicillin-binding protein 3 (PBP3), an important component of bacterial cell wall synthesis [46]. A notable finding of the present study was the predominance of these mutations among environmental water isolates, with only two veterinary isolates carrying the same mutation profile. This observation supports the hypothesis that aquatic environments may function as reservoirs of resistant *A. baumannii* clones. The absence of these mutations in isolates from cattle (sheep and goats) and poultry (turkey and guinea fowl) indicates that chromosomal fluoroquinolone resistance was not uniformly distributed across host species in this collection. This finding highlights the heterogeneous distribution of resistance mechanisms among different ecological niches.

Genes associated with tolerance to non-antibiotic stressors were also widely detected. The *merA*, *merD*, *merR*, and *merT* genes were identified in 74.6% of the isolates, while *nreB* and the disinfectant-associated efflux determinant *amvA* were highly conserved. Heavy-metal resistance determinants have been discussed in relation to the co-occurrence and possible co-selection of antimicrobial resistance genes in bacterial populations [47]. However, heavy-metal concentrations and disinfectant exposure were not measured in the present study. The presence of these genes therefore indicates genomic potential for tolerance to environmental stressors but does not directly demonstrate active heavy-metal-mediated co-selection. The observed differences in antimicrobial resistance profiles should be interpreted with consideration of the unequal distribution of sequence types between the two isolate sources. The predominance of ST52 among veterinary isolates and ST2 among environmental isolates may partially contribute to the observed resistance patterns, and therefore these differences likely reflect both isolate source and lineage-associated characteristics.

All major *A. baumannii* virulence factors were detected in the genomes of the investigated isolates. Genes involved in biofilm formation, surface adherence, iron acquisition, lipopolysaccharide biosynthesis, quorum sensing, pili assembly, phospholipase production, and secretion systems were widely conserved among veterinary and environmental isolates. The presence of *bap* and the *csu* operon is particularly relevant because biofilm formation and surface attachment contribute to the persistence of *A. baumannii* on biotic and abiotic surfaces [48]. Biofilm-associated growth can also reduce antimicrobial penetration and enhance bacterial survival under adverse conditions [49]. Its matrix, phenotypic heterogeneity, and spatial organization promote antimicrobial tolerance and horizontal gene transfer, making biofilms reservoirs for AMR [50]. The limited variation in virulence gene distribution between veterinary and environmental isolates suggests that both populations retain a broadly conserved set of determinants associated with colonization, persistence, and environmental adaptation. Genes associated with iron acquisition and bacterial competition were also widely represented. The acinetobactin-associated *bas* and *bau* loci were highly conserved across the collection. Iron acquisition is important for the survival and pathogenicity of *A. baumannii*, particularly under iron-limited conditions encountered within the host [51]. Similarly, the detection of type VI secretion system genes, including *tss* components, *hcp*, and *vgrG/tssl,* indicates the presence of genetic machinery associated with interbacterial competition and adaptation to heteromicrobial environments [52]. The core virulence determinants of our study closely match the study reports of clinical strains, in particular, outer membrane proteins and biofilm-associated genes detected in over 90% of the clinical strains and 52% of the strains, respectively [53,54], which underscores the similar virulence nature of our non-clinical *A. baumannii* with clinical strains. These VFG systems may contribute to the persistence of *A. baumannii* in complex ecological settings and may improve survival in polymicrobial environments such as wastewater and animal-associated habitats. Additionally, the high prevalence of conserved virulence loci indicates that even isolates with comparatively lower antimicrobial resistance may retain substantial pathogenic potential [55], which does not necessarily indicate gene expression or increased pathogenicity. Detection of multiple virulence-associated genes and clinically relevant ST2 suggests that these isolates possess genomic features commonly associated with pathogenic *A. baumannii*. However, the genomic detection of virulence-associated genes alone does not establish their expression or functional activity, and further phenotypic studies are required to determine their contribution to the behavior of the isolates examined here.

MLST revealed a restricted population structure consisting of only ST52 and ST2. ST52 was the predominant lineage, accounting for 74.6% of the total isolate collection and 97.2% of veterinary isolates. In contrast, ST2 represented 58% of environmental isolates and was identified in only two veterinary isolates. ST2 is a globally distributed *A. baumannii* lineage frequently associated with antimicrobial resistance and healthcare-associated infections [56]. Its predominance among environmental isolates in the present study is therefore important, particularly because the ST2-associated population carried *blaOXA-23*, *armA*, and several additional acquired resistance determinants. The global dissemination and genomic evolution of multidrug-resistant ST2 populations have been well documented [56,57]. In comparison, the predominance of ST52 among veterinary isolates in the present collection indicates a distinct lineage distribution within the sampled animal population.

The close relationship between lineage and resistance gene content was further supported by the population genomic analyses. The cgMLST separated ST2 and ST52 into distinct genetic groups, while several isolates within each lineage shared identical allelic profiles or differed by only a few core-genome alleles. Core-genome SNP analysis similarly showed limited within-lineage diversity, with pairwise differences ranging from 0 to 7 SNPs in both ST2 and ST52. The low SNP distances and close phylogenetic clustering indicate the presence of highly related isolates within each lineage. Comparable whole-genome approaches have been used to identify closely related ST2 populations and investigate their persistence and transmission [58]. In the present study, closely related isolates were recovered from different animal hosts and environmental sources. Our findings reveal close genetic relatedness among *A. baumannii* isolates recovered from environmental and veterinary sources, suggesting the possibility of shared reservoirs or a circulation area. Rather, the findings demonstrate the occurrence of closely related *A. baumannii* genotypes across the sampled veterinary and environmental settings.

The entry of livestock-derived fecal microbes into aquatic ecosystems poses a significant challenge to the One Health concept. Samples collected from animals and from rivers used for irrigation and livestock rearing have revealed shared microbial communities, indicating microbial exchange between livestock and aquatic environments through activities such as animal washing and swimming [59].

Pan-genome analysis further supported the lineage structure identified by MLST, cgMLST, and SNP analysis. Of the 4204 gene clusters identified, 73.9% belonged to the core-genome, indicating a substantial conserved genomic component across the isolate collection. In contrast, approximately one-quarter of the pan-genome consisted of shell genes, and the gene presence–absence matrix showed distinct accessory gene patterns between ST2 and ST52. Genomic plasticity and variation in accessory gene content are recognized features of *A. baumannii* and can contribute to the adaptation and diversification of individual lineages [60]. The distinct accessory genome patterns observed between ST2 and ST52 therefore indicate that lineage-associated genomic differences extend beyond core-genome variation. These findings also provide a genomic context for the distinct resistance gene profiles observed between the two sequence types.

Plasmid prediction identified contigs with high nucleotide similarity to previously described *Acinetobacter* plasmids, including plasmids reported in *A. baumannii* ATCC 19606 and other *A. baumannii* strains, as well as pSGAir0122 from *A. schindleri* [60,61]. Plasmid-associated sequence matches were identified among isolates from different veterinary hosts and environmental water samples. Mobile genetic elements are important contributors to genome plasticity in *A. baumannii*, and complete genome analysis of strain ATCC 19606 has demonstrated the presence of diverse mobile genetic elements [62]. However, the present plasmid analysis was based on predicted plasmid-associated contigs from short-read genome assemblies and sequence similarity searches. Consequently, complete plasmid structures and the physical association of individual antimicrobial resistance genes with these plasmids could not be established. Long-read sequencing and hybrid genome assembly would be required to resolve the complete plasmid architecture and determine their genetic cargo.

Together, the findings demonstrate that the veterinary and environmental *A. baumannii* populations examined in this study share a broad background of intrinsic resistance and virulence-associated determinants but differ in their lineage composition. ST52 predominated among veterinary isolates, whereas ST2 was enriched among environmental isolates and carried a distinct pattern of acquired resistance genes. At the same time, cgMLST and SNP analyses identified highly related isolates across different hosts and environmental sources. The close correspondence between sequence type, resistance gene distribution, and accessory genome structure highlights the need to consider bacterial population structure when comparing antimicrobial resistance across ecological reservoirs. These findings support continued genomic and AMR surveillance for monitoring clonal distribution, identifying the emergence and persistence of high-risk lineages across animal and environmental sources within a One Health framework.

## 4. Materials and Methods

### 4.1. Study Design and Sample Collection

This observational study was conducted in Guntur and Prakasam districts of Andhra Pradesh, India, to investigate the genomic diversity and AMR profiles of *A. baumannii* recovered from veterinary and environmental sources. Veterinary specimens were collected from livestock and poultry in Guntur district, whereas environmental water samples were collected from the Krishna River and its associated rivulets in the neighboring Prakasam district. The selected river stretches supply water to the border villages of Guntur district, where it is extensively used for livestock watering and agricultural activities, thereby providing an ecologically relevant environmental source for comparison with veterinary isolates.

Veterinary samples were collected from livestock (cattle, buffaloes, sheep, and goats) and poultry (chicken, guinea fowl, and turkey) presented to veterinary healthcare facilities in the study region. The specimens included milk, uterine discharge, urine, dung, nasal discharge, cloacal swabs, and poultry droppings, depending on the animal species and clinical presentation. All specimens were collected aseptically, transported under refrigerated conditions, and processed immediately upon arrival at the laboratory.

Environmental water sampling was carried out at five locations along the main course of the Krishna River, with adjacent sampling sites separated by approximately 10 km. An additional seven sampling sites were selected from adjoining rivulets located approximately 20 km from the main river. These sites were selected because river and rivulet water are routinely used for irrigation and livestock husbandry in the surrounding villages. Water samples were collected aseptically using sterile sampling bottles, transported under refrigerated conditions, and processed for microbiological analysis.

A total of 104 veterinary specimens and 52 environmental water samples were analyzed. Following microbiological isolation and species confirmation, 122 non-duplicate *A. baumannii* isolates, comprising 72 veterinary isolates and 50 environmental isolates, were isolated and subjected to antimicrobial susceptibility testing, whole-genome sequencing, and comparative genomic analyses. Out of 72 veterinary isolates included in the present study, 69 isolates were reported in the previous study [63], which included apparently healthy animals exclusively. The remaining three isolates included in the current study were recovered from infected animals and were not part of the previous report. Details of sample sources and identification numbers are provided in (Supplementary Material S1).

### 4.2. Isolation and Identification of A. baumannii

The collected samples were serially diluted (10^−4^ to 10^−6^) and cultured on HiCrome *Acinetobacter* Agar Base (HiMedia Laboratories, Mumbai, India). Plates were incubated aerobically at 37 °C for 18–24 h, and presumptive *Acinetobacter* colonies were purified by repeated subculturing to obtain pure isolates with a single morphology. Species identification was performed using matrix-assisted laser desorption/ionization time-of-flight mass spectrometry (MALDI-TOF MS; Bruker Daltonics, Bremen, Germany). Only isolates confirmed as *A. baumannii* (Bruker score ≥ 2.0) were included for subsequent antimicrobial susceptibility testing and whole-genome sequencing.

### 4.3. Antimicrobial Susceptibility Testing and Phenotypic Detection of Resistance Mechanisms

All confirmed *A. baumannii* isolates were subjected to antimicrobial susceptibility testing (AST) using the MICRONAUT-S MDR MRGN-Screening system (Bruker Daltonics GmbH & Co. KG, Bremen, Germany) according to the manufacturer’s instructions. The MICRONAUT-S MDR MRGN panel comprised antimicrobial agents representing the major clinically relevant antimicrobial classes, including penicillins, β-lactam/β-lactamase inhibitor combinations, cephalosporins, carbapenems, fluoroquinolones, aminoglycosides, glycylcyclines, polymyxins, sulfonamides, amphenicols and phosphonic acid derivatives. The panel also included inhibitor-based combinations for phenotypic screening of carbapenemase-associated resistance profiles according to the manufacturer’s recommendations (Table S1).

Fresh overnight cultures grown on blood agar were used for inoculum preparation. Morphologically similar colonies were suspended in sterile 0.9% saline and adjusted to a turbidity equivalent to a 0.5 McFarland standard (approximately 1.5 × 10^8^ CFU/mL). The bacterial suspension was subsequently diluted in cation-adjusted Mueller–Hinton broth (CAMHB) and inoculated into the MICRONAUT microdilution plates according to the manufacturer’s recommendations. Plates were incubated aerobically at 35–37 °C for 18–24 h, and minimum inhibitory concentrations (MICs) were determined by photometric evaluation using the MICRONAUT software (version 6).

Phenotypic screening of resistance mechanisms was performed using the inhibitor-based combinations integrated within the MICRONAUT-S MDR MRGN panel. Interpretation was based on inhibitor-associated MIC changes according to the manufacturer’s interpretation algorithm implemented in MICRONAUT software.

For data analysis, *A. baumannii* was selected as the taxon, and MICs were interpreted, and isolates were categorized as susceptible, intermediate, or resistant according to the CLSI M100-Ed33, Clinical and Laboratory Standards Institute (CLSI), USA-2023 guidelines. *Escherichia coli* ATCC 25922 was included as the quality-control strain throughout the study.

### 4.4. Whole-Genome Sequencing

Genomic DNA was extracted from bacterial cultures grown in Luria–Bertani (LB) broth at 37 °C for 18 h. Cells were harvested by centrifugation at 10,000× *g* for 2 min, and genomic DNA was extracted using the High Pure PCR Template Preparation kit (Roche Diagnostics GmbH, Mannheim, Germany) according to the manufacturer’s instructions. DNA quality and concentration were assessed using a Qubit4 fluorometer (Thermo Fisher Scientific, Waltham, MA, USA) with the Qubit dsDNA HS Assay Kit. Sequencing libraries were prepared using the Nextera XT DNA library preparation kit (Illumina, San Diego, CA, USA) followed by paired-end whole-genome sequencing on the Illumina MiSeq platform (Illumina, USA).

Raw sequencing reads were assessed for quality using FastQC v0.12.1 (https://www.bioinformatics.babraham.ac.uk/projects/fastqc, accessed on 10 August 2025). The reads were *de novo* assembled using Shovill v1.0.4 (https://github.com/tseemann/shovill, accessed on 14 March 2025), and annotation was performed using Bakta v1.8.2 with database v5.0, followed by assembly quality assessment using QUAST v5.2.0. Raw sequencing data have been deposited in the European Nucleotide Archive (ENA) under BioProject accession PRJEB89272.

### 4.5. Genomic Characterization

Assembled genomes were analyzed to identify AMR determinants, virulence-associated genes, sequencing types, and plasmid-associated contigs. Acquired AMR genes were identified using ABRicate v1.0.1 (https://github.com/tseemann/abricate, accessed on 14 March 2025) against the Comprehensive Antibiotic Resistance Database (CARD) [64]. Chromosome point mutations associated with antimicrobial resistance were detected using AMRFinderPlus v3.11.26 (https://github.com/ncbi/amr, accessed on 24 March 2025) with the species-specific -O option for *A. baumannii* and the NCBI AMR reference gene database. Virulence-associated genes were identified using ABRicate v1.0.1 (https://github.com/tseemann/abricate, accessed on 28 March 2025) with the Virulence Factor Database (VFDB) [65] at thresholds of ≥80% sequence identity and coverage.

In silico MLST was performed using mlst v2.23.0 (https://github.com/tseemann/mlst, accessed on 14 April 2025) based on the Pasteur typing scheme. Plasmid-associated contigs were predicted using Platon v1.7 (https://github.com/oschwengers/platon, accessed on 20 April 2025) [66] and were further examined by sequence similarity searches against the NCBI BLAST database to identify the closest homologous plasmid sequences [67].

### 4.6. Comparative Genomic Analysis

Comparative genome analyses were performed to investigate the genetic relatedness and population structure of the *A. baumannii* isolates. Core-genome MLST (cgMLST) and subsequent minimum spanning tree analysis were carried out using Ridom Seqsphere + v8.2.0 (Ridom GmbH, Münster, Germany) [68,69]. Core-genome SNP analysis was performed using Snippy v4.6.0 (https://github.com/tseemann/snippy, accessed on 20 April 2025) and pairwise SNP differences were calculated using snp-dists v0.8.2 (https://github.com/tseemann/snp-dists, accessed on 30 April 2025).

Maximum-likelihood phylogenetic trees were reconstructed from the core SNP alignment using RAxML) v8.2.12 with the GTRGAMMA model of nucleotide substitution with gamma-distributed rate heterogeneity [70], and the resulting tree was visualized using Microreact (https://microreact.org/, accessed on 30 April 2025) [71].

Pan-genome analysis was performed using Panaroo v1.4.2 [72]. The genome annotation was performed using Bakta v1.8.2 with database v5.0. Based on the filtered core gene alignment, a phylogenetic tree was calculated using RAxML v8.2.12 with the GTRGAMMA model of rate heterogeneity. The pan-genome composition and corresponding phylogenetic relationships were visualized using Phandango (https://jameshadfield.github.io/phandango/, accessed on 10 May 2026) [73].

### 4.7. Statistical Analysis

Descriptive statistics were used to summarize antimicrobial susceptibility profiles, resistance determinants, virulence-associated genes, and sequence type distributions. Differences in the distribution of MDR and XDR phenotypes and the major sequence types ST52 and ST2 between veterinary and environmental isolates were evaluated using two-sided Fisher’s exact tests [74]. Odds ratios (ORs) and corresponding 95% confidence intervals were calculated to estimate the strength of association.

Differences in antimicrobial resistance between veterinary and environmental isolates were assessed separately for each antimicrobial agent using two-sided Fisher’s exact tests. For these comparisons, isolates categorized as resistant were compared with non-resistant isolates, comprising susceptible and intermediate categories. The prevalence of individual virulence-associated genes was similarly compared between the two isolate sources using two-sided Fisher’s exact tests based on binary gene presence–absence data. To account for multiple comparisons across antimicrobial agents and virulence-associated genes, *p*-values were adjusted using the Benjamini–Hochberg false discovery rate (FDR) procedure.

The differences in virulence gene composition between veterinary and environmental isolates were further evaluated by permutational multivariate analysis of variance (PERMANOVA) based on Jaccard distances calculated from the binary virulence gene presence–absence matrix [75]. Statistical significance was assessed using 9999 permutations. An FDR-adjusted *p*-value < 0.05 was considered statistically significant for multiple comparisons, and a two-sided *p*-value < 0.05 was considered significant for individual tests. Statistical analyses were performed using Python (Jupyter Notebook application (version 7.5.5 within the Anaconda Python environment)) with SciPy statmodels [76] and scikit-bio packages.

## 5. Limitations

This study did not include clinical isolates to correlate with the One Health aspect. The second limitation of the study is the absence of a host–pathogen interaction study. Also, the study was conducted in a single time-point perspective rather than a time-point study, which limits the information on temporal evolution of ARGs and VFGs. The other limitation of the study includes the absence of transcriptomic or functional validation.

## 6. Conclusions

This study provides a comparative genomic characterization of *A. baumannii* isolates from veterinary and environmental sources in Andhra Pradesh, India. A high prevalence of MDR and XDR phenotypes was observed, together with a broad collection of intrinsic and acquired antimicrobial resistance determinants. The isolate population was dominated by two sequence types with distinct source distributions: ST52 predominated among veterinary isolates, whereas ST2 was more frequent among environmental isolates. The distribution of several acquired resistance genes closely followed this lineage structure, indicating that differences in resistance gene profiles between the two sources are strongly influenced by the underlying *A. baumannii* population composition.

Despite these differences, veterinary and environmental isolates shared a largely conserved set of virulence-associated and intrinsic resistance determinants. cgMLST and core-genome SNP analyses further identified highly related isolates within ST2 and ST52 across different hosts and environmental sources, while pan-genome analysis revealed distinct accessory gene patterns between the two lineages. The findings demonstrate the importance of considering lineage structure when interpreting antimicrobial resistance across ecological sources and support continued genomic surveillance of *A. baumannii* at the animal-environment interface. Further studies incorporating human *A. baumannii*, functional enrichment analysis, longitudinal sampling, epidemiological data, and long-read sequencing will be required to clarify transmission pathways and resolve the contribution of mobile genetic elements to the dissemination of antimicrobial resistance.

## Figures and Tables

**Table 1:** Antimicrobial susceptibility profiles of *A. baumannii* isolates.

Antimicrobial Class	Antimicrobial Agent	Susceptible *n* (%)	Intermediate *n* (%)	Resistant *n* (%)
Fluoroquinolone	Ciprofloxacin (CIP)	71 (58.2)	1 (0.8)	50 (41.0)
	Levofloxacin (LEV)	70 (57.4)	0 (0.0)	52 (42.6)
Aminoglycoside	Amikacin (AK)	72 (59.0)	1 (0.8)	49 (40.2)
Polymyxin	Colistin (COL)	1 (0.8)	121 (99.2)	0 (0.0)
Amphenicol	Chloramphenicol (CMP)	0 (0.0)	0 (0.0)	122 (100.0)
Phosphonic acid derivative	Fosfomycin (FOS)	0 (0.0)	0 (0.0)	122 (100.0)
Folate pathway inhibitor	Trimethoprim/Sulfamethoxazole (T/S)	24 (19.7)	0 (0.0)	98 (80.3)
Penicillin	Piperacillin (PIP)	0 (0.0)	0 (0.0)	122 (100.0)
β-lactam/β-lactamase inhibitor combination	Piperacillin/Tazobactam	67 (54.9)	3 (2.5)	52 (42.6)
Third-generation cephalosporin	Cefotaxime (CTX)	0 (0.0)	0 (0.0)	122 (100.0)
	Ceftazidime (CAZ)	70 (57.4)	0 (0.0)	52 (42.6)
Cephalosporin/β-lactamase inhibitor combination	Ceftazidime/Avibactam (CAA)	71 (58.2)	0 (0.0)	51 (41.8)
	Ceftolozane/Tazobactam (CTA)	71 (58.2)	1 (0.8)	50 (41.0)
Carbapenem	Imipenem (IMP)	69 (56.6)	0 (0.0)	53 (43.4)
	Meropenem (MER)	69 (56.6)	3 (2.5)	50 (41.0)

MIC interpretations were performed according to the Clinical and Laboratory Standards Institute (CLSI) M100 guidelines (2023). Colistin susceptibility was interpreted according to the current CLSI recommendations for *A. baumanni*.

## Data Availability

Selected isolates (*n* = 18) have been deposited in the NCMR bacterial repository, India. Raw sequencing data for the isolates have been deposited in the European Nucleotide Archive under BioProject PRJEB89272. The genomic datasets are deposited in the Mendeley data repository (https://doi.org/10.17632/64db2fz8vv.1).

## Supplementary Materials

The following supporting information can be downloaded at: https://www.mdpi.com/article/10.3390/ijms27177571/s1.
