# Supplementary material for: Whole-Genome Analysis Reveals Antimicrobial Resistance and Population Structure of Environmental and Veterinary Acinetobacter baumannii
Source: Int J Mol Sci. 2026 Aug 24;27(17):7571. doi: 10.3390/ijms27177571 (PMC13566364; doi:10.3390/ijms27177571)
Supplement: Supplementary file 1 [file ijms-27-07571-s001.zip › Supplementary Tables.pdf]

Supplementary Table S1. Antimicrobial agents, inhibitor-based combinations, and concentrations included in the MICRONAUT-S MDR MRGN-Screening panel.

Sl. No.	Antibiotic Class	Antimicrobial agent/combination	Abbreviation	Concentration (µg/mL)	Purpose
1	Penicillins	Piperacillin	PIP	8–16	AST
2	Penicillin + β-lactamase inhibitors	Piperacillin/Tazobactam	PIT	4/4–8/4	AST
3	Cephalosporins	Cefotaxime	CTX	1–2	AST
		Ceftazidime	CAZ	1–4	AST
4	Cephalosporin + inhibitor combinations	Ceftazidime/3-APB	CZB	0.25–4	Phenotypic AmpC detection
	Cephalosporin + β-lactamase inhibitor	Ceftazidime/Avibactam	CAA	8/4	AST
		Ceftolozane/Tazobactam	CTA	1/4	AST
5	Carbapenems	Imipenem	IMP	1–4	AST
		Meropenem	MER	0.125–4	AST
6	Carbapenem + inhibitor combinations	Meropenem/3-APB	MEB	0.25–1	Phenotypic KPC detection
		Meropenem/EDTA	MEE	0.25–1	Phenotypic MBL detection
7	Monobactam derivative	Temocillin	TMO	128	Phenotypic OXA-48-like detection
8	Fluoroquinolones	Ciprofloxacin	CIP	0.25	AST
		Levofloxacin	LEV	0.5	AST
9	Aminoglycosides	Amikacin	AMK	4–16	AST
10	Polymyxins	Colistin	COL	1–4	AST
11	Sulfonamides	Trimethoprim/Sulfamethoxazole	T/S	1/19-4/76	AST
12	Amphenicols	Chloramphenicol	CMP	16	AST
13	Phosphonic acid derivatives	Fosfomycin	FOS	128	AST

Supplementary Table S2. Resistance classification of each isolate (MDR/XDR).

Sl. No.	Isolate ID	ST type	Name	Source	Resistance Classification
1	22RX25205	52	CL12	Veterinary	MDR
2	22RX25206	52	CL02		
3	22RX25207	52	CL15		
4	22RX25208	52	CL16		
5	22RX25209	52	CL09		
6	22RX25210	52	CL11		
7	22RX25211	52	FS 1		
8	22RX25212	52	FS 2		
9	22RX25213	52	FS 3		
10	22RX25214	52	FS 4		
11	22RX25215	52	FS 5		
12	22RX25216	52	MS2		
13	22RX25217	52	MS5		
14	22RX25218	52	MS 7		
15	22RX25219	52	MS 8		
16	22RX25220	52	MS 9B		
17	22RX25221	52	MS 10		
18	22RX25222	52	MS 11A		
19	22RX25223	52	MS 12		
20	22RX25224	52	MS 13A		
21	22RX25225	52	MS 13D		
22	22RX25226	52	MS 13E		
23	22RX25227	52	MS 14		
24	22RX25228	52	MS 15		
25	22RX25229	52	MS 23		
26	22RX25230	52	MS 24		
27	22RX25231	52	MS 25		
28	22RX25232	52	MS 26		
29	22RX25233	52	MS4		
30	22RX25234	52	MS 16		
31	22RX25235	52	MS 17		
32	22RX25236	52	MS 19		
33	22RX25237	52	MS 20		
34	22RX25238	52	GMS 1		
35	22RX25239	52	GMS 2		
36	22RX25240	52	GMS 2B		
37	22RX25241	52	SMS 2		
38	22RX25242	52	SMS4		

39	22RX25243	52	SMS 4B		
40	22RX25244	52	SMS 6		
41	22RX25245	52	SMS 7		
42	22RX25246	52	NDS 1		
43	22RX25247	52	NDS 2		
44	22RX25248	52	NDS 5		
45	22RX25249	52	NDS 5B		
46	22RX25250	52	PDS 2B		
47	22RX25251	52	PDS 3		
48	22RX25252	52	PDS 4A		
49	22RX25253	52	US8		
50	22RX25254	52	US10		
51	22RX25255	52	UPS 1 A		
52	22RX25256	52	UPS 4A		
53	22RX25257	52	UPS 5A		
54	22RX25258	52	UPS 6		
55	22RX25259	52	UPS 7		
56	22RX25260	52	UPS 8A		
57	22RX25261	52	UPS 9B		
58	22RX25262	52	UPS 9D		
59	22RX25263	2	UPS 10B		
60	22RX25264	2	KAS1B	Environmental	
61	22RX25266	52	KAS1C		
62	22RX25267	52	KAS2A		
63	22RX25268	52	KAS2B		
64	22RX25269	52	KAS2C		
65	22RX25270	52	KAS3A		
66	22RX25271	52	KAS3B		
67	22RX25272	52	KPS3B		
68	22RX25273	52	KGOLS2A		
69	22RX25274	52	KGOLS2B		
70	22RX25275	52	KGOLS2C		
71	22RX25276	52	KGUNS2C		
72	22RX25277	52	PDS 1	Veterinary	XDR
73	22RX25278	52	PDS 2A		
74	22RX25279	52	PDS 4B		
75	22RX25280	52	US 1		
76	22RX25281	52	US3		
77	22RX25282	52	US4		
78	22RX25283	52	US7		
79	22RX25284	52	UPS 2A		
80	22RX25285	52	UPS 2B		
81	22RX25286	52	WND 1		

82	22RX25287	2	WND 2		
83	22RX25288	2	WND 3		
84	22RX25289	2	WND4		
85	22RX25290	2	KAS1A	Environmental	
86	22RX25291	2	KAS3C		
87	22RX25292	2	KPS1A		
88	22RX25293	2	KPS1B		
89	22RX25294	52	KPS1C		
90	22RX25295	2	KPS2A		
91	22RX25296	2	KPS2B		
92	22RX25297	2	KPS2C		
93	22RX25298	2	KPS3A		
94	22RX25299	2	KPS3C		
95	22RX25300	2	KVS1A		
96	22RX25301	2	KVS1B		
97	22RX25302	2	KVS1C		
98	22RX25303	52	KVS2A		
99	22RX25304	52	KVS2B		
100	22RX25305	52	KVS2C		
101	22RX25306	52	KVS3A		
102	22RX25307	52	KVS3C		
103	22RX25308	52	KGOLS1A		
104	22RX25309	52	KGOLS1B		
105	22RX25310	2	KGOLS1C		
106	22RX25311	2	KGOLS3B		
107	22RX25312	2	KGOLS3C		
108	22RX25313	2	KGUNS1A		
109	22RX25314	2	KGUNS1B		
110	22RX25315	2	KGUNS1C		
111	22RX25316	52	KGUNS2A		
112	22RX25317	2	KGUNS3A		
113	22RX25318	2	KGUNS3B		
114	22RX25319	2	KGUNS3C		
115	22RX25320	2	WS1		
116	22RX25321	2	WS2		
117	22RX25322	2	WS3		
118	22RX25323	2	WS 4A		
119	22RX25324	2	WS5		
120	22RX25325	52	WS 6A		
121	22RX25326	52	WS 6B		
122	22RX25327	52	WS 7		

Supplementary Table S3. Distribution of antimicrobial resistance, heavy metal resistance, and disinfectant resistance genes detected among the 122 *Acinetobacter baumannii* isolates.

Resistance category	Gene	Resistance mechanism	Veterinary (n = 72)	Environmental (n = 50)	Total (n = 122)	Frequency (%)
Aminoglycosides	armA	16S rRNA methyltransferase	2	29	31	25.4
	aph(3'')-Ib	Aminoglycoside phosphotransferase	2	29	31	25.4
	aph(6)-Id	Aminoglycoside phosphotransferase	2	29	31	25.4
	ant(3'')-IIa	Aminoglycoside nucleotidyltransferase	72	50	122	100
Tetracyclines	tet(B)	Efflux pump	2	29	31	25.4
Sulfonamides	sul2	Altered dihydropteroate synthase	70	21	91	74.6
Macrolides	mph(E)	Macrolide phosphotransferase	2	29	31	25.4
	msr(E)	ABC-F ribosomal protection protein	2	29	31	25.4
β-lactams	blaADC-2	AmpC β-lactamase	70	21	91	74.6
	blaADC-73	AmpC β-lactamase	2	29	31	25.4
	blaADC-158	AmpC β-lactamase	70	21	91	74.6
	blaOXA-23	Carbapenem-hydrolysing class D β-lactamase	2	29	31	25.4
	blaOXA-66	Intrinsic class D β-lactamase	2	29	31	25.4
	blaOXA-98	Class D β-lactamase	70	21	91	74.6
Multidrug efflux systems	AbaQ	MFS efflux pump	72	50	122	100
	abeM	MATE family efflux pump	72	50	122	100
	abeS	SMR family efflux pump	72	50	122	100
	abaF	Major facilitator superfamily (MFS) efflux pump	72	50	122	100
	adeA	AdeABC RND efflux pump	72	50	122	100
	adeB	AdeABC RND efflux pump	72	50	122	100
	adeC	AdeABC outer membrane channel	2	29	31	25.4

	adeF	AdeFGH RND efflux pump	72	50	122	100
	adeG	AdeFGH RND efflux pump	72	50	122	100
	adeH	AdeFGH outer membrane protein	72	50	122	100
	adeI	AdeIJK RND efflux pump	72	50	122	100
	adeJ	AdeIJK RND efflux pump	72	50	122	100
	adeK	AdeIJK outer membrane channel	72	50	122	100
	adeL	Transcriptional regulator (AdeFGH)	72	50	122	100
	adeN	TetR family regulator (AdeIJK)	70	21	91	74.6
	adeR	Response regulator (AdeRS)	72	50	122	100
	adeS	Sensor kinase (AdeRS)	72	50	122	100
Heavy metal resistance	merA	Mercuric reductase	70	21	91	74.6
	merD	Mercury operon coregulator	70	21	91	74.6
	merR	Mercury-responsive transcriptional regulator	70	21	91	74.6
	merT	Mercury transport protein	70	21	91	74.6
	nreB	Nickel efflux transporter	72	50	122	100
Disinfectant resistance	amvA	Multidrug efflux pump	72	50	122	100

Supplementary Table S4. List of ARGs with acquired AMR genes and known chromosomal resistance-associated mutation.

**Category**	**Resistance determinant**	**Target**	**Resistance mechanism**	**Typical mode of acquisition**
**Acquired resistance gene**	***blaOXA-23***	OXA-23 carbapenemase	Hydrolysis of β-lactam ring	Horizontal gene transfer (transposons/plasmids/chromosome)
	blaOXA-66	Intrinsic OXA-51-like oxacillinase	β-lactam hydrolysis	Chromosomal
	blaOXA-98	OXA-51-like oxacillinase variant	β-lactam hydrolysis	Chromosomal

	blaADC-73	Acinetobacter-derived cephalosporinase	Cephalosporin hydrolysis	Chromosomal
	blaADC-158	AmpC cephalosporinase	Cephalosporin hydrolysis	Chromosomal
	armA	16S rRNA methyltransferase	Ribosomal methylation	Plasmids/transposons
	aph(3″)-Ib	Aminoglycoside phosphotransferase	Drug inactivation	Horizontal transfer
	aph(6)-Id	Aminoglycoside phosphotransferase	Drug inactivation	Horizontal transfer
	ant(3″)-IIa	Aminoglycoside nucleotidyltransferase	Drug adenylation	Horizontal transfer
	tet(B)	Tetracycline efflux pump	Active efflux	Plasmids/transposons
	sul2	Altered dihydropteroate synthase	Target replacement	Plasmids
	mph(E)	Macrolide phosphotransferase	Drug inactivation	Horizontal transfer
	msr(E)	ABC-F ribosomal protection protein	Ribosomal protection	Horizontal transfer
	abaF	Fosfomycin efflux transporter	Active efflux	Chromosomal
	adeA, adeB, adeC	*AdeABC* RND efflux pump	Active multidrug efflux	Chromosomal
	adeF, adeG, adeH	*AdeFGH* efflux pump	Active multidrug efflux	Chromosomal
	adeI, adeJ, adeK	AdeIJK efflux pump	Active multidrug efflux	Chromosomal

	adeL	LysR regulator	Regulates AdeFGH expression	Chromosomal
	adeN	TetR regulator	Regulates AdeIJK	Chromosomal
	adeR, adeS	Two-component regulatory system	Activates AdeABC	Chromosomal
	abeM	MATE efflux pump	Drug efflux	Chromosomal
	abeS	SMR efflux pump	Drug efflux	Chromosomal
	AbaQ	Major facilitator superfamily transporter	Drug efflux	Chromosomal
	amvA	Major facilitator transporter	Drug efflux	Chromosomal
**Chromosomal mutation associated with resistance**	***gyrA S81L***	DNA gyrase A	Altered quinolone target	Point mutation
	parC S84L	Topoisomerase IV	Altered quinolone target	Point mutation
	ftsI A515V	Penicillin-binding protein 3	Reduced β-lactam binding	Point mutation
	Mutations in adeR/adeS *(if detected)*	Two-component regulator	Constitutive efflux pump activation	Point mutation
	Mutations in adeL *(if detected)*	LysR regulator	Increased AdeFGH expression	Point mutation
	Mutations in adeN *(if detected)*	TetR regulator	Increased AdeIJK expression	Point mutation

Supplementary Table S5. Chromosomal point mutation detected using AMRFinderPlus.

Gene	Amino acid substitution	No. of isolates (%)	Associated antibiotic/resistance

gyrA	S81L	31 (25.4%)	Fluoroquinolones (ciprofloxacin, levofloxacin)
parC	S84L	31 (25.4%)	Fluoroquinolones
ftsI	A515V	31 (25.4%)	Encodes penicillin-binding protein 3, or PBP3

Supplementary Table S6. Chromosomal association of virulence genes detected in *A. baumannii.*

Virulence system	Genes	Typical genomic location
Quorum sensing	*abaI, abaR*	Chromosome
AdeFGH efflux pump	*adeF, adeG, adeH*	Chromosome
Outer membrane protein	*ompA*	Chromosome
Biofilm (PNAG synthesis)	*pgaA, pgaB, pgaC, pgaD*	Chromosome
Csu pili	*csuA/BABCDE*	Chromosome
Type IV pili	*pilA, pilB, pilC, pilE, pilF, pilG, pilH, pilI, pilJ, pilM, pilN, pilO, pilP, pilQ, pilR, pilS, pilT, pilU, pilV, pilW, pilX, pilY1, fimT, fimU, fimV, tsaP*	Chromosome
Type II secretion system	*gspC, gspD, gspE1, gspE2, gspF, gspG, gspH, gspI, gspK, gspL, gspM, gspN, gspO (pilD)*	Chromosome
Type VI secretion system (T6SS)	*tssA, tssB, tssC, tssE, tssF, tssG, tssK, tssL, tssM, hcp (tssD), vgrG (tssI), clpV (tssH), tagX*	Chromosome
Acinetobactin biosynthesis	*basA, basB, basC, basD, basF, basG, basH, basI, basJ*	Chromosome
Acinetobactin transport	*bauA, bauB, bauC, bauD, bauE, bauF*	Chromosome
Haem utilization	*hemO*	Chromosome
Enterobactin synthesis	*entE*	Chromosome
Phospholipases	*plc1, plc2, plcD*	Chromosome
Lipooligosaccharide synthesis	*lpxA, lpxB, lpxC, lpxD, lpxL, lpxM, lpsB*	Chromosome
Protein glycosylation	*pgi, galE, galU*	Chromosome
Penicillin-binding protein	*pbpG*	Chromosome
BarAB two-component system	*barA, barB*	Chromosome
Capsule-associated genes	*tviB*	Chromosome
ACICU_RS loci	*ACICU_RS00395–ACICU_RS16840*	Chromosome
Autotransporter adhesins	*bap, ata*	Chromosome

Supplementary Table S7. Predicted plasmid-associated contigs and their closest plasmid sequence matches identified by NCBI BLAST analysis.

Plasmid hit	Number of isolates	Query coverage and identity	ST	Host
Acinetobacter baumanni ATCC 19606 = CIP 70.34 = JCM 6841 strain ATCC 19606 plasmid pMAC complete sequence	23	52%; 99.8%	52	Guinea Fowl, Chicken, Turkey, Buffalo, Cow, Goat, Sheep, Water
Acinetobacter baumanni strain AR_0052 plasmi unnamed4	28	83%; 99.8%	52	Goat, Water
Acinetobacter schindleri strai SGAir0122 plasmi pSGAir0122, complet sequence	23	45%; 98.7%	52	Guinea Fowl, Chicken, Turkey, Buffalo, Cow, Goat, Sheep, Water
Acinetobacter schindleri strai SGAir0122 plasmi pSGAir0122, complet sequence	28	55%; 99.8%	52	Buffalo<br>Goat<br>Water
Acinetobacter baumanni strain AB3927 plasmi pAB3927, complet sequence	18	98%; 98.9%	52	Water
Acinetobacter baumanni ATCC 19606 DNA complete genome	17	100%; 99.8%	52	Buffalo, Water
Acinetobacter baumanni ATCC19606 plasmi pATCC19606-1 DNA complete sequence	22	52%; 100%	52	Chicken, Cow, Water
Acinetobacter baumanni strain B20AB01 plasmi pB20AB01a, complet sequence	23	100%; 100%	2	Buffalo
			52	Water
Acinetobacter baumanni strain A85 plasmid pA85-1b complete sequence	10	55%; 99.8%	52	Water
Acinetobacter baumanni strain G20AB05 plasmi pG20AB05	24	100%; 100%	2	Water
